# Detection of *Shigella* in Milk and Clinical Samples by Magnetic Immunocaptured-Loop-Mediated Isothermal Amplification Assay

**DOI:** 10.3389/fmicb.2018.00094

**Published:** 2018-02-06

**Authors:** Liding Zhang, Qiujiang Wei, Qinqin Han, Qiang Chen, Wenlin Tai, Jinyang Zhang, Yuzhu Song, Xueshan Xia

**Affiliations:** ^1^Molecular Medicine Research Center of Yunnan Province, Faculty of Life Science and Technology, Kunming University of Science and Technology, Kunming, China; ^2^Kunming Biomed International, Kunming, China; ^3^Yunnan Molecular Diagnosis Centre, The Second Affiliation Hospital of Kunming Medical University, Kunming, China

**Keywords:** *Shigella*, IC-LAMP, immunocapture, detection, specificity

## Abstract

*Shigella* is an important human food-borne zoonosis bacterial pathogen, and can cause clinically severe diarrhea. There is an urgent need to develop a specific, sensitive, and rapid methodology for detection of this pathogen. In this study, loop-mediated isothermal amplification (LAMP) combined with magnetic immunocapture assay (IC-LAMP) was first developed for the detection of *Shigella* in pure culture, artificial milk, and clinical stool samples. This method exhibited a detection limit of 8.7 CFU/mL. Compared with polymerase chain reaction, IC-LAMP is sensitive, specific, and reliable for monitoring *Shigella.* Additionally, IC-LAMP is more convenient, efficient, and rapid than ordinary LAMP, as it is more efficiently enriches pathogen cells without extraction of genomic DNA. Under isothermal conditions, the amplification curves and the green fluorescence were detected within 30 min in the presence of genomic DNA template. The overall analysis time was approximately 1 h, including the enrichment and lysis of the bacterial cells, a significantly short detection time. Therefore, the IC-LAMP methodology described here is potentially useful for the efficient detection of *Shigella* in various samples.

## Introduction

*Shigella* is recognized as an important bacterial pathogen worldwide, and may cause several diseases, such as purulent blood, diarrhea, spasms, and shocks, which leads a huge threat to society (Iseki et al., 2007). There are about 1.8 million patients died for diarrhea and a majority of these cases (160 million cases per year) have been attributed to *Shigella* (Lauri et al., 2011).

Studies have revealed the association between *Shigella* and most of the bacillary dysentery in developing nations (Iseki et al., 2007; Lin et al., 2010). *Shigella* is also one of the necessary inspection items for laboratory monkey export. Therefore, for use in the food industry and to protect public health, rapid, specific, and sensitive methodologies are required to detect this pathogen (Wang et al., 2015). Although culture-based techniques are considered the gold standard for the detection of *Shigella* in a variety of samples, the culture and enrichment of *Shigella* are still needed, followed by confirmed with serological and biochemical assay, requiring about 7 days, in a time-consuming and labor-intensive process (Mokhtari et al., 2012). All kinds of studies have reported that the large invasion plasmid is existed in all *Shigella*. Besides, the *ipaH* gene is present both on the invasion plasmid and on the chromosome of *Shigella*. Therefore, the *ipaH* has been used for the target gene to detect *Shigella* in many studies. Based on *ipaH*, *Shigella* can be screened accurately among numerous plasmids. Use of an *ipaH* gene, the time to detect *Shigella* from stool samples has been decreased to 0.5 h from the typical 4–7 days using the traditional approach. Later, the *ipaH* gene combined with polymerase chain reaction (PCR) was developed to shorten assay time. However, this assay has low sensitivity and usually requires spending a few hours enriching the bacterial, before extraction of genomic DNA. In addition, a thermal cycling machine, an electrophoresis apparatus and a gel imaging equipment are also needed (Liew et al., 2014). In another approach, the *ipaH* gene targeted real-time PCR assay was applied for detection of *Shigella* in rectal swab specimens. This assay is rapid and highly sensitive, but also has not yet become common in hospital laboratories due to the requirement for specific equipment.

A novel nucleic acid amplification method, loop-mediated isothermal amplification (LAMP), was recently described by Notomi et al. (2000). LAMP is a simple, rapid, highly specific, and sensitive detection methodology, and has been successfully employed in the past for the detection of various pathogens, including parasites, fungi, bacteria, and viruses (Vaagt et al., 2013; Huang et al., 2014; Law et al., 2014; Ye et al., 2014; Song et al., 2016). Furthermore, the method does not need any special equipment, as it will appears a white precipitate in the positive reaction can be judged by the naked eye, facilitating use of this method as a field test with simple and cost-effective reaction equipment. However, there are still some limits to use of this method, including the need to extract genomic DNA or plasmids for use as templates and difficulty in applying this technology to a large number of samples.

The current study sought to develop and validate a diagnostic IC-LAMP-based methodology for real-time and visual detection of the *Shigella*. The LAMP combined with magnetic immunocapture technology (IC-LAMP), which integrates both the specificity of antibody and LAMP, and was more efficiently enrich pathogen cells and does not require the extraction of genomic DNA.

## Materials and Methods

### Strains Used in This Study

The *Shigella* strain used in this study was isolated from monkey by Kunming Biomed International. *Escherichia coli*, *Staphylococcus aureus*, *Salmonella*, *Listeria*, and *Pseudomonas aeruginosa* were used for specificity testing as shown in **Supplementary Table S1**.

### Preparation of Polyclonal Antibody against *Shigella*

*Shigella* was used to produce antibodies in BALB/c mice. Prior to immunization, a blood sample from each mouse was collected. The blood was incubated for 1 h at 37°C and was then centrifuged at 3000 rpm for 20 min at 4°C. The serum was gathered as a negative control. Mice were first immunized by back multipoint subcutaneous injection using a mixture of 100 μL (10^8^ inactivated bacteria) of diluted *Shigella* with an equal volume of Freund’s complete adjuvant. After an interval of 2 weeks, injections were given with a mixture of 100 μL of diluted *Shigella* in incomplete Freund’s adjuvant. Subsequently, the blood was obtained 1 week after the last injection, the serum was collected as described above, and polyclonal antibody specific for *Shigella* was produced. Animal experiments and procedures were performed ethically according to the Guidelines for Animal Experiments and approved by the Committee on Experimental Animals at Kunming University of Science and Technology.

### Titration of Polyclonal Antibody

Indirect enzyme-linked immunosorbent assay (ELISA) was used to determine the titer of the serum. The cultured *Shigella* (inactivated) was diluted to a concentration of 10^8^ CFU/mL in phosphate-buffered saline (PBS; 2.7 mmol/L KCl, 137 mmol/L NaCl, 2 mmol/L KH_2_PO_4,_ 10 mmol/L Na_2_HPO_4,_ pH 7.4) and then was coated on 96-well plates at 100 μL and incubated for 3 h at 37°C. The coated wells were washed three times with PBS containing 0.05% Tween-20 and then blocked with 100 μL of blocking buffer (5% skimmed milk in PBS-T) for 2 h at 37°C. Followed by three washes with PBS-T buffer, the coated wells were incubated with 100 μL of serum against *Shigella* with different dilutions (from 1:200 to 1: 409600) at 37°C for 2 h. After incubation, the coated wells were washed three times with PBS-T buffer and assayed using the goat anti-mouse IgG (H+L) HRP (GenScript, United States) as a secondary antibody. The optical density of each well was then measured at 450 nm using a microplate reader (Bio-Rad) (Tang et al., 2016).

### Bacteriostatic Experiment of Polyclonal Antibody

To ensure that the hyperimmune serum of *Shigella* could recognize and capture the bacterium, a bacteriostatic experiment was performed. The cells were cultured overnight and then were diluted to concentrations of 10^8^, 10^6^, and 10^4^ CFU/mL with sterile water. Then, 50 μL of bacterial liquid culture was applied to LB solid medium (0.5% Yeast Extract, 1% Tryptone, 1.5% Agar, 1% NaCl, pH 7.0) as a blank control. Separately, 50 μL bacteria culture plus 2 μL multiple antiserum or 50 μL bacterial culture with 2 μL negative serum (from mice that were not immunized with *Shigella*) were also applied to LB solid medium as positive and negative controls, respectively. The agar plates were cultured at 37°C for 12 h. After culturing, the inhibitory effect of anti-*Shigella* serum on the growth of different concentrations of *Shigella* was measured.

### Genomic DNA Extraction

The genomic DNA of all bacteria used in this study was extracted by a Bacteria Genomic DNA kit (Zomanbio, China). The DNA concentration was measured using an ultraviolet spectrophotometer and stored at -20°C before use.

### PCR Reaction

The outer primers of the LAMP assay (F3: 5′-ATACCGTCTCTGCACGCA-3′ and B3: 5′-GCCTTCTGATGCCTGATGG-3′) were used to perform PCR reaction in a 25 μL volume which contained 12.5 μL of 2 × TSINGKE Master Mix (containing DNA polymerase 1 U, 1.5 mM MgCl_2_, 200 mM each dNTP), 10 μM of each primer, and 50 ng genomic DNA. The reactions were carried out in GeneAmp PCR System 9700 with the following amplification conditions: pre-denaturing at 95°C for 5 min, followed by 34 cycles of denaturing at 95°C for 30 s, annealing at 57°C for 30 s, and extending at 72°C for 30 s, and a final extension at 72°C extension for 7 min. A total of 5 μL of the PCR product was analyzed by electrophoresis in 1% agarose gel.

### The LAMP Assay

The LAMP assay was performed as described by Notomi et al. (2000) and Savan et al. (2004). LAMP requires a set of four primers (B3: 5′-GCCTTCTGATGCCTGATGG-3′, F3:5′-ATACCGTCTCTGCACGCA-3′, BIP:5′-CTTTCGCTGTTGCTGCTGATGCTTTTCCGGAGATTGTTCCATGTGA-3′, and FIP:5′-CTGTCGAAGCTCCGCAGAGGTTTTGGATTC CGTGAACAGGTCG-3′), recognizing six distinct target sites of the DNA sequence. Primers used for the LAMP reaction were designed using the primer design software Primer Explorer V5, primer software available on the Net laboratory website^1^, and targeted the conserved C-terminal region of the *ipaH* gene. Primers FIP and BIP were composed of the complementary sequence of the F1, B2, and the direct sequence of F2, B1, respectively. Primers F3 and B3 were located outside the F2 and B2 regions, respectively. The LAMP reaction was performed in a total volume of 25 μL of a mixture containing 1 μL DNA template, 20 pM each of the FIP and BIP primers, 2.5 pM each of the F3 and B3 primers, 1 μL of Bst DNA polymerase (Eiken Chemical Co., Ltd., Tokyo, Japan), 2.5 μL of 10 × LAMP buffer (200 mmol/L Tris–HCl, 100 mmol/L (NH_4_)_2_SO_4_, 100 mmol/L KCl, 1% Triton X-100, pH 8.8), 150 nM Mg^2+^, 40 nM dNTP mixture, and 9.5 μL nuclease-free water. The reaction of the mixture was initiated by an incubation of 63°C for 50 min and terminated at 80°C for 2 min. A total of 5 μL of the product was analyzed by separation on 3% agarose gel.

### Preparation of Immunomagnetic Beads

Protein A/G-coated magnetic beads (Biotool, United States) were suspended, and transferred from 25 μL to a 1.5 mL tube. The magnetic beads were washed extensively with binding buffer (150 mmol/L NaCl, 50 mmol/L Tris, 0.5% Tween-20, pH 7.5). Then, magnetic separation was performed, the supernatant was removed and 200 μL of fresh binding buffer was added. The mouse anti*-Shigella* serum was diluted (1:4,000) into 5% nonfat dry milk in PBS containing 0.05% Tween-20. A total of 50 μL of mouse anti-*Shigella* serum was transferred into the 1.5 mL tubes and incubated for 2 h at 37°C. Magnetic separation was performed again. The nonbinding anti-*Shigella* antibody in tubes was then removed and the beads were washed three times by PBS-T.

### The Standard IC-LAMP Assay

After preparation of immunomagnetic beads, the immunomagnetic beads were used to capture *Shigella* at 37°C for 0.5 h. Then, 10 μL solution A (125 mM NaOH, 1 mM EDTA, 0.1% Tween-20) was added to the tubes, followed by incubation at 65°C for 10 min to lyse the bacteria cells. Finally, 10 μL solution B (125 mM HCl, 10 mM Tris) was added to terminate the reaction. Then, 1–6 μL of bacteria lysate was used as template for LAMP amplification as described above.

### Specificity and Sensitivity of LAMP, IC-LAMP, and PCR

In order to test the specificity of the designed primers, we amplified the genomic DNA of five common food-borne pathogenic bacteria collected in this study by using PCR, LAMP, and IC-LAMP.

The constructed templates were used to detect the sensitivity of the PCR, LAMP, and IC-LAMP. The plasmid concentration was calculated using the deduced polynomial model described as:

$$C=\frac{X\times 10^{-9}}{(A+Y)\times 660}\times N_{A}$$

### Practical Application of IC-LAMP for the Detection of *Shigella* in Milk

In order to test the applicability of IC-LAMP technology in milk, *Shigella* was mixed with pasteurized milk samples, purchased from a grocery store in Kunming and identified as *Shigella*-negative by traditional culture techniques and PCR method. First, in order to check the minimal detectable colony forming units (CFUs), the cultures of *Shigella* strains were serially diluted (10^-1^–10^-9^). The CFUs of the plate were counted after 24 h at 37°C. Next, the aliquots of 100 μL appropriate dilutions with *Shigella* were inoculated into the milk samples, for final concentrations of *Shigella* of approximately 8.7 × 10^6^, 8.7 × 10^5^, 8.7 × 10^4^, 8.7 × 10^3^, 8.7 × 10^2^, 8.7 × 10^1^, and 8.7 × 10^0^ CFU/mL. The milk samples were transferred to a tube, which containing magnetic beads, antibody, and incubated at 37°C for 30 min. After enrichment of the cells, the tubes were washed three times with PBS-T, followed by the addition of 10 μL of solution A and incubation at 65°C for 10 min. Finally, 10 μL of solution B was added to terminate the reaction. Then, 1–6 μL of bacteria lysate was used for IC-LAMP. A non-contaminated milk sample was used as a negative control. Each milk sample was tested five times with the IC-LAMP.

### Detection of *Shigella* in Monkey Clinical Samples by IC-LAMP Assay

*Shigella* screening is a necessary component of inspection for laboratory monkey export. We tested direct detection of *Shigella* in monkey clinical samples from feces and/or anal swabs. To do this, 46 clinical samples were collected from monkeys for IC-LAMP assay detection and analysis. These samples were first suspended in 1 mL sterile LB liquid medium and incubated at room temperature for 1 h, before the suspension was transferred to 1.5 mL tubes that contained magnetic beads and antibody. All tubes were reacted at 37°C for 0.5 h. After enrichment of the cells, the tubes were washed three times with PBS-T, and then 10 μL of solution A was added and the mixture was incubated at 65°C for 10 min. Next, 10 μL of solution B was added to terminate the reaction. After this treatment, 1–6 μL of bacteria lysate was used for IC-LAMP. A non-contaminated clinical sample was used as a negative control.

### Isolation and Identification of *Shigella* in Monkey Clinical Samples by Traditional Methods

In the animal room experimental area, a cotton swab was used to take a small amount of fresh manure. The middle of the stool was used for the sample to avoid urine, food residue, or other contaminants. As an alternative, anal swabs were taken from primates if fresh feces were not available.

The swabs or anal swabs with sticky feces were seeded on a petri dish using a zoning method. After inoculation, the dish was inverted (agar plate bottom up), numbered, and cultured at 37°C for 18–24 h. Care was taken to avoid scratching the surface of the medium when applying the samples.

There were colorless, round, flat, or slightly raised colonies that were smooth and moist, with neat edges, and about 2 mm in diameter. *Shigella* colonies are generally larger, translucent, and sometimes may appear flat, with an irregular edge of a rough type of colony.

In the biological safety cabinet, an inoculation ring was used to pick 2–3 suspicious colonies that were then cultured on a Triple Sugar Iron Agar Medium at 37°C for 18–24 h. If the selected colonies were *Shigella*, the slope does not produce acid, so the bottom of the acid does not produce gas, and the hydrogen sulfide is negative.

Onto a clean glass slide, a small amount of *Shigella* multivalence diagnostic serum (Lanzhou Institute of Biological Products Co., Ltd, China) was applied. The inoculation ring was then used to take Salmonella–Shigella (SS) agar plates or Triple Sugar Iron Agar Medium and add them to the serum, at a ratio of 1:1 to measure agglutination. All manipulations were performed under sterile conditions.

## Results

**FIGURE 1:**
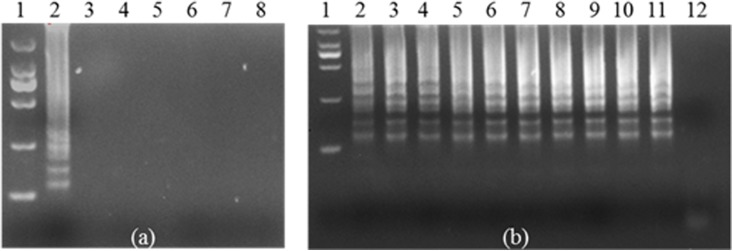
Specificity and sensitivity of *ipaH*-LAMP assays using the genome of *Shigella* strains and the plasmid containing the *ipaH* gene of *Shigella* strains. **(a)** 3% agarose gel electrophoresis of the *Shigella*-templated LAMP Product; lane 1, 2000 bp DNA marker; lane 2, positive LAMP products; lane 3 to lane 7 represent LAMP reactions of genomic DNA of *Escherichia coli*, *Staphylococcus aureus*, *Salmonella*, *Listeria*, and *Pseudomonas aeruginosa*, and lane 8 is the reaction with nuclease-free water as a negative control. **(b)** The sensitivity assay. Lane 1, 2000 bp DNA marker. Lanes 2 through 11, positive plasmid used as template at concentration of 10^0^ to 10^9^ copies per reaction. Nuclease-free water was used as a negative control in lane 12.

In this study, *Shigella* was used to produce antibodies in BALB/c mice. After four immunizations, polyclonal antibody was successfully prepared. The antibody titer was approximately 1: 409600 by ELISA (**Supplementary Figure S1J**). A bacteriostatic experiment was performed to ensure that the polyclonal antibody of *Shigella* was used to recognize and capture the bacterium (**Supplementary Figures S1A–I**). Two pairs of primers were designed and used for IC-LAMP. First, the outer primers (F3, B3) were used to test specificity (**Supplementary Figure S2a**) and sensitivity (**Supplementary Figure S2b**). Next, a set of four primers (F3, B3, FIP, and BIP) was used for the LAMP assay. Two pairs of primers could specifically amplify the *ipaH* gene of *Shigella*, but not for the other five bacterial strains (**Figure 1a**). The sensitivity test of LAMP showed that the amplified fragments were obviously observed in samples of recombinant plasmid (**Figure 1b**).

**FIGURE 2:**
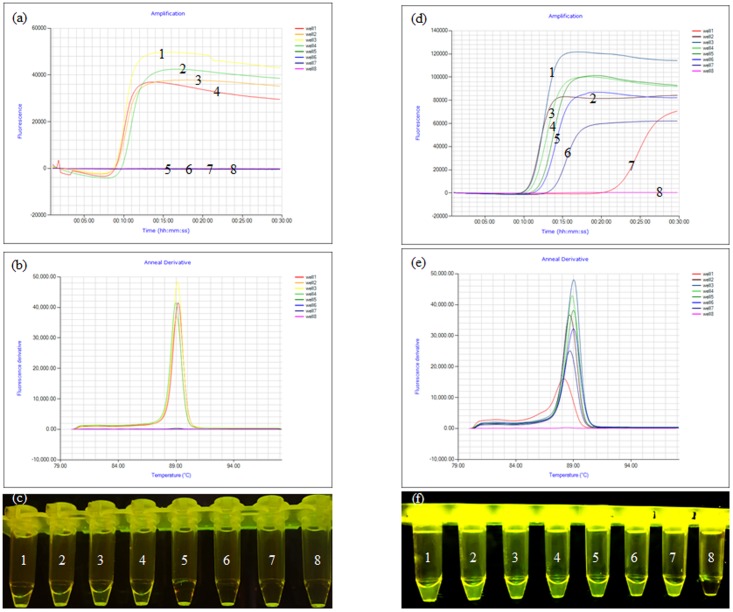
Specificity and sensitivity of the *ipaH*-IC-LAMP assay. **(a)** Amplification curve of the specificity of IC-LAMP assays, **(b)** annealing curve of the specificity of IC-LAMP assays, **(c)** specificity of IC-LAMP assay observed under ultraviolet lamp; tubes 1, 2, 3, and 4 contained lysates of mixed bacteria with *Shigella* (*Escherichia coli*, *Shigella*, *Staphylococcus aureus*, *Salmonella*, *Listeria*, and *Pseudomonas aeruginosa*); tubes 5, 6, and 7 contained lysates of mixed bacteria without *Shigella* (*E. coli*, *S. aureus*, *Salmonella*, *Listeria*, and *P. aeruginosa*); tube 8 was a negative control. **(d)** Amplification curve of the sensitivity of IC-LAMP assays, **(e)** annealing curve of the sensitivity of IC-LAMP assays, **(f)** the sensitivity of the IC-LAMP assay as observed under ultraviolet lamp, tubes 1, 2, 3, 4, 5, 6, and 7 represent 8.7 × 10^6^, 8.7 × 10^5^, 8.7 × 10^4^, 8.7 × 10^3^, 8.7 × 10^2^, 8.7 × 10^1^, and 8.7 × 10^0^ CFU/ml, respectively, and tube 8 was a negative control.

Lysates containing mixed bacteria (*E. coli*, *Shigella*, *S. aureus*, *Salmonella*, and *P. aeruginosa*) were captured by immunomagnetic beads (immunomagnetic beads were obtained by coupling the antibody to magnetic spheres). Lysates containing mixed bacteria, but not *Shigella* (*E. coli*, *S. aureus*, *Salmonella*, and *P. aeruginosa* only) were captured by immunomagnetic beads to determine the specificity of the assay. The results showed that the amplification curves and the green fluorescence were obviously observed only when the mixed bacteria contained *Shigella* (**Figures 2a–c**). The *Shigella* was serially diluted into skim milk (from 8.7 × 10^6^ to 8.7 × 10^0^ CFU/ml) to test the sensitivity of using the IC-LAMP assay in milk. The results showed that the amplification curve and the green fluorescence were obviously observed even at a concentration of only 8.7 × 10^0^ CFU/ml. Compared with PCR and LAMP, the IC-LAMP method was much faster and more sensitive for detection (**Figures 2d–f**).

To further verify the accuracy of IC-LAMP, 46 clinical samples were subjected to IC-LAMP. The results revealed that of the 46 clinical samples, 12 were positive for the presence of *Shigella*, and 34 were negative. The clinical samples were also subjected to traditional biochemical identification and the serum agglutination test. These tests also indicated that there were 12 positive and 34 negative samples in the 46 monkey stool samples. DNA was extracted from the 46 clinical samples using TIANamp Stool DNA Kit (TIANGEN, China), and then was subjected to LAMP assay. The results of the regular LAMP assay indicated that there were 8 positive and 38 negative samples in the 46 monkey stool samples. Thus, compared with LAMP, IC-LAMP can more accurately, quickly, and specifically detect *Shigella* in monkey clinical samples (**Table 1**).

**Table 1:** Comparison between IC-LAMP, LAMP, and traditional biochemical identification and serum agglutination test.

Results	Methods		
	
	IC-LAMP	LAMP	Thegold standard test^∗^
Positive	12	8	12
Negative	34	38	34
Total	46	46	46


*^∗^Selective medium isolation and commercial Shigella multivalence diagnostic serum identification as the gold standard test.*

## Discussion

*Shigella* has various hosts, and can be scattered among all kinds of food and animals. However, a rapid and precise diagnosis method for detection of *Shigella* is still limited. Molecular techniques can help species identification, but current techniques of PCR, LAMP, culture-based techniques, and biochemical assays have limitations, precluding wider application of these methods in field laboratories and in under-resourced settings (Wang et al., 2014). As a novel isothermal detection technology, LAMP is a simple, rapid, highly specific, and sensitive detection methodology but it does not efficient enrich cells, making it difficult to apply this technology to special samples that may have low concentrations of pathogens, such as milk, feces, and seafood.

In this study, LAMP was combined with magnetic IC-LAMP for the detection of *Shigella* in processed milk and clinical samples (**Supplementary Figure S3**). This method is different from regular LAMP, and does not require enrichment of *Shigella* before detection, so can capture *Shigella* in processed milk and clinical samples. In this fast detection system, two sets of primers of the LAMP were specifically designed to the target *ipaH* gene of *Shigella*. Under isothermal conditions, the IC-LAMP methodology was able to detect the pathogen in real-time in a reaction, and the detection and amplification reaction could be conducted simultaneously within 1 h including enrichment of *Shigella* (Wang et al., 2015).

The species-specific gene *ipaH* present in all *Shigella* was used as the target sequence to design the specific primers of the IC-LAMP. The specificity of IC-LAMP reactions was good, with positive results generated in the assay of *Shigella* strains but not for non-*Shigella* strains. The established IC-LAMP assay exhibited a detection limit of 8.7 × 10^0^ CFU/mL in the processed milk. The IC-LAMP assay established here was also used to detect *Shigella* in clinical samples. The results of assaying 46 clinical samples by IC-LAMP matched the conclusions from culture-based techniques and biochemical assays. Overall, the IC-LAMP assay can detect *Shigella* accurately and quickly.

## Conclusion

A reliable method to monitor *Shigella* was developed using LAMP combined with magnetic immunocapture assay (IC-LAMP). This study demonstrated the practicality of this method for the detection of *Shigella* from processed milk and clinical samples. With the one-step procedure, even large-scale examination can be performed within 1 h. Furthermore, the magnetic IC-LAMP, simplified DNA extraction method, and portable LAMP equipment allow this method to be applied for on-site detection for detection of *Shigella.*

## Author Contributions

LZ and QW designed and drafted the work. LZ, QW, QH, QC, and WT performed the experiments, analyzed the data, and interpreted the results. JZ, YS, and XX designed the work and revised it critically.

## Conflict of Interest Statement

The authors declare that the research was conducted in the absence of any commercial or financial relationships that could be construed as a potential conflict of interest.

## Footnotes

1 http://primerexplorer.jp/e/v5_manual/index.html

## Supplementary Material

**FIGURE S1 |** Bacteriostatic experiment and titer of *anti-Shigella* serum by ELISA. **(A–C)** Represent inoculation of 50 uL⋅10^8^cfu/mL, 10^6^cfu/mL, and 10^4^cfu/mL of *Shigella*, respectively; **(D–F)** represent inoculation of 50 uL⋅10^8^ cfu/mL, 10^6^cfu/mL, and 10^4^cfu/mL of *Shigella* plus 2 uL negative serum, respectively; **(G–I)** represent inoculation of 50 uL⋅10^8^cfu/mL, 10^6^cfu/mL, and 10^4^cfu/mL of *Shigella* plus 2 uL of antiserum, respectively. **(J)** presents the titer of mice serum at different dilution ratios as determined by ELISA.

**FIGURE S2 |** Specificity and sensitivity of the *ipaH-PCR* assays using the genome of *Shigella* strains and the plasmid containing the *ipaH* gene of *Shigella* strains. **(a)** 1% agarose gel electrophoresis of the *Shigella*-templated PCR product; lane 1, 2000 bp DNA marker; lane 2, positive PCR products; lane 3 to lane 7 represent the genome of *Escherichia coli, Staphylococcus aureus, Salmonella, Listeria*, and *Pseudomonas aeruginosa* PCR products, lane 8 nuclease-free water as a negative control. **(b)** 1% agarose gel electrophoresis of the *Shigella*-templated PCR product; lane 1, 2000 bp DNA marker; lane 2 to lane 11 represent different levels of plasmid (10^9^-10^0^ copies), lane 12 nuclease-free water as a negative control.

**FIGURE S3 |** Schematic diagram of IC-LAMP.

**TABLE S1 |** Bacterial strains used in this study.
