# Supplementary figures and images for: Detection of Shigella in Milk and Clinical Samples by Magnetic Immunocaptured-Loop-Mediated Isothermal Amplification Assay
Source: Front Microbiol. 2018 Feb 6;9:94. doi: 10.3389/fmicb.2018.00094 (PMC5807921; doi:10.3389/fmicb.2018.00094)

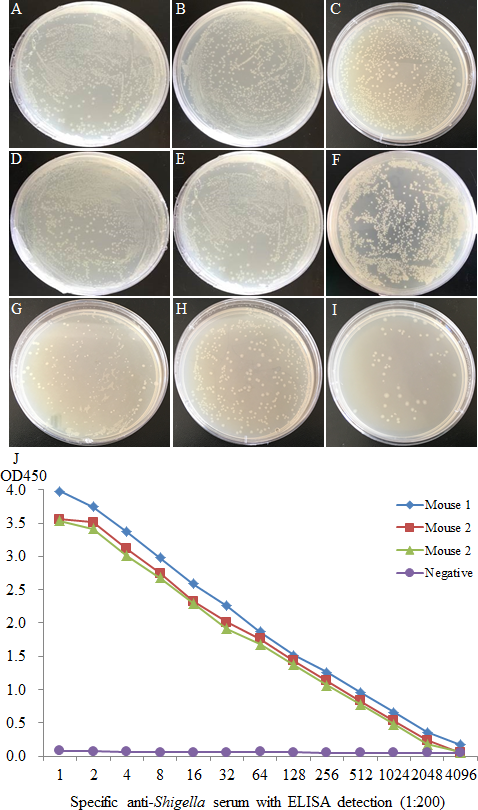

Supplement: Supplementary file 1 [file Image_1.tif]

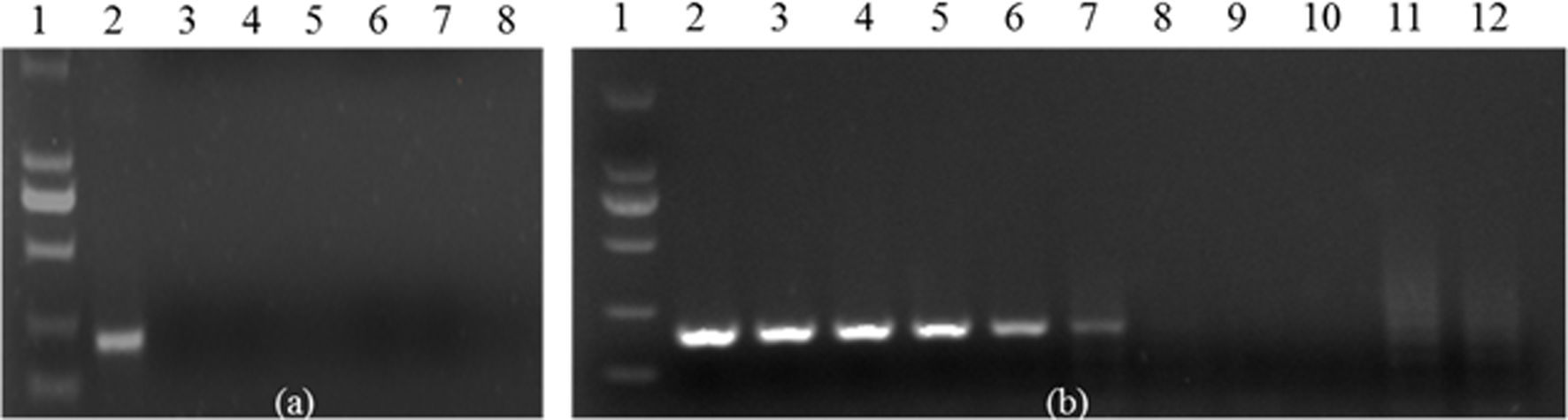

Supplement: Supplementary file 2 [file Image_2.tif]

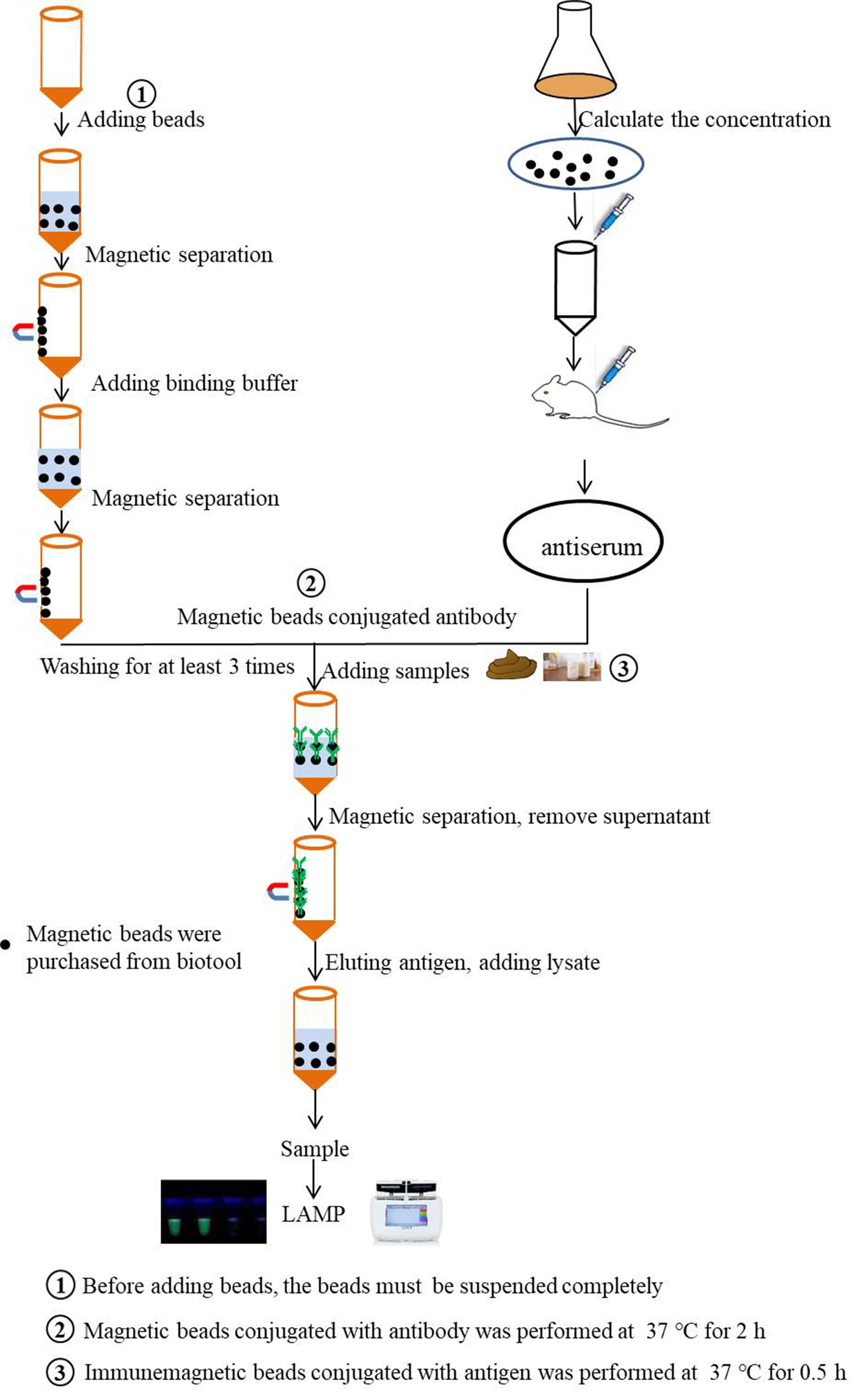

Supplement: Supplementary file 3 [file Image_3.TIF]
