# Supplementary material for: Detection of Shigella in Milk and Clinical Samples by Magnetic Immunocaptured-Loop-Mediated Isothermal Amplification Assay
Source: Front Microbiol. 2018 Feb 6;9:94. doi: 10.3389/fmicb.2018.00094 (PMC5807921; doi:10.3389/fmicb.2018.00094)
Supplement: Supplementary file 4 [file Table_1.DOCX]

| Table S1 Bacterial strains used in this study |  |
| --- | --- |
| Bacterial | Bacterial strains Source |
| *Shigella*(1606SH192) | Isolate from monkey |
| *Pseudomonas aeruginosa* (1606SE0010) | Isolate from scretion substance |
| *Escherichia coli* | ATCC25922 |
| *Staphylococcus aureus* | ATCC29213 |
| *Salmonella* | ATCC8739 |
| *Listeria* (1608SE0080) | Isolate from scretion substance |
